# Paediatric Langerhans Cell Histiocytosis: A 20-Year Single-Centre Retrospective Study of 35 Cases

**DOI:** 10.7759/cureus.99421

**Published:** 2025-12-16

**Authors:** Faliq Abdullah, Clare Carpenter, Madeleine Adams, Andrew Bamber

**Affiliations:** 1 Trauma and Orthopaedics, University Hospital of Wales, Cardiff, GBR; 2 Paediatric Oncology, University Hospital of Wales, Cardiff, GBR; 3 Cellular Pathology, University Hospital of Wales, Cardiff, GBR

**Keywords:** bone lesions, braf mutation, craniofacial bones, langerhans cell histiocytosis, multisystem disease, paediatric oncology, pediatric bone tumors, retrospective study, single-system disease, skeletal involvement

## Abstract

Background

Langerhans cell histiocytosis (LCH) is a rare proliferative disorder of bone marrow-derived histiocytes that most often affects children. Skeletal involvement is the most frequent manifestation of paediatric LCH, but the lesions can mimic malignancy or infection, posing a diagnostic challenge. We present a retrospective case series of 35 paediatric patients with LCH, highlighting skeletal lesion patterns, multisystem involvement, treatment approaches, and clinical outcomes.

Methodology

Medical records and imaging of 35 paediatric patients diagnosed with LCH between 2005 and 2025 in Wales were retrospectively reviewed. Clinical data, including age, sex, site of disease, radiological characteristics, immunohistochemical markers, treatment regimens, and outcomes, were collected and analysed. Disease was classified as unifocal, multifocal, or multisystem. Diagnosis was based on clinical, radiological, and histological findings. Histopathological confirmation was obtained where available, using CD1a, S100, CD68, and Langerin in selected cases. In patients without a biopsy, diagnosis was based on characteristic clinical and radiological features.

Results

The cohort had a median age of four years (range: 19 days to 16 years), with 22 males (62.9%) and 13 females (37.1%). Skeletal involvement occurred in 30 (85.7%) of 35 patients, most commonly affecting the skull (22 cases, 62.9%), followed by the femur, scapula, spine, pelvis, sternum, clavicle, rib, humerus, ulna, and tibia. All lesions were purely lytic, with most appearing ill-defined; two lesions were well-circumscribed. Extra-skeletal involvement occurred in eight patients, affecting the pituitary, skin, lymph nodes, liver, spleen, lungs, and bone marrow. Immunohistochemistry was performed in 28 cases, with most showing positivity for CD1a, S100, and CD68. Ten patients were diagnosed clinically without histology. Genetic testing identified BRAF mutations in four of seven tested patients.

Management was based on disease extent. Nineteen patients with unifocal disease were managed conservatively with observation, biopsy, or curettage. All patients with multifocal or multisystem disease received systemic chemotherapy. Patients treated after 2021 received the LCH-IV protocol, and those treated before 2017 received LCH-III. All 35 patients were alive at the last follow-up. Eight experienced disease relapse, primarily at skeletal sites.

Conclusions

Skeletal involvement in paediatric LCH most often presents as ill-defined lytic lesions in the skull and other flat bones, although any bone may be affected. Diagnosis requires careful clinicoradiological assessment and histopathological confirmation when available. Most patients in this cohort had single-system, unifocal disease and achieved excellent outcomes with conservative or systemic therapy as appropriate. Multisystem disease remains more challenging, but favourable outcomes can still be achieved with protocol-based treatment. Early recognition, multidisciplinary evaluation, and long-term follow-up are essential due to the risk of relapse and potential late sequelae.

## Introduction

Langerhans cell histiocytosis (LCH) is an uncommon paediatric disorder characterised by the clonal accumulation of CD1a-positive, langerin-positive dendritic cells in various tissues [1]. The disease has a broad clinical spectrum, ranging from isolated bone lesions to life-threatening multisystem involvement. LCH is rare, with an incidence of roughly 4-8 cases per million children per year [1]. It most often presents in early childhood (median age ~3-4 years) and shows a slight male predominance [1].

LCH is clinically classified as single-system (SS) disease, where only one organ or system is involved, or multisystem (MS) disease with multiple organs affected [1]. Bone (skeletal system) is the most frequently involved organ system in children, affected in about 80% of paediatric LCH cases [1]. Consequently, many patients have what was historically called eosinophilic granuloma - an isolated bone lesion - or multifocal bone disease without other organ involvement. Other organs commonly involved in LCH include the skin (cutaneous lesions), lymph nodes, liver, spleen, and endocrine organs such as the pituitary gland. Patients with multisystem LCH, especially those involving risk organs (liver, spleen, or bone marrow), are considered high-risk and require systemic therapy [1].

The skeletal manifestations of LCH can affect virtually any bone. Notably, there is a predilection for the axial skeleton and flat bones [2]. More than half of paediatric LCH bone lesions occur in the skull (craniofacial bones) [2,3]. Other frequently involved bones are the proximal long bones (such as the femur), ribs, vertebrae, and pelvis [2]. Clinically, children with bone LCH often present with localized pain, swelling, or a pathologic fracture at the lesion site [4]. Because LCH bone lesions can radiologically mimic infection or malignancy, biopsy confirmation is usually required for diagnosis [5]. Histopathology classically reveals Langerhans cells with grooved nuclei, accompanied by eosinophils and other inflammatory cells, and immunohistochemistry (IHC) is positive for CD1a and S100 proteins [5].

This article reviews our experience with 35 paediatric patients diagnosed with LCH, including detailed analysis of the 30 patients who had skeletal involvement. We describe the anatomical distribution of bone lesions, the distinction between unifocal and multifocal disease, and the management strategies employed. We also compare our findings with those in the literature to provide context. By characterising skeletal involvement patterns and outcomes, we aim to aid clinicians in recognising typical bone lesion sites and formulating appropriate management plans for paediatric LCH.

## Materials and methods

Methodology

We conducted a retrospective review of paediatric patients diagnosed with LCH between 2005 and 2025 at our tertiary care centre. Inclusion criteria were age younger than 16 years at diagnosis and clinical, radiological, or histological diagnosis of LCH, including patients with isolated extra-skeletal involvement.

Of the 35 patients included, histopathological confirmation was obtained in 27 cases (77.1%), demonstrating typical Langerhans cell phenotype markers (predominantly CD1a, CD68, S100, and occasionally Langerin) [6]. The remaining 8 patients (22.8%) were diagnosed clinically and radiologically, primarily representing earlier cases diagnosed before 2012 without biopsy. Clinical data, including age, sex, sites of involvement, lesion characteristics, IHC results, treatment strategies, and patient outcomes, were extracted from medical records. Radiological findings (X-ray, MRI, or CT) were reviewed to document skeletal lesion characteristics, noting specifically their lytic and circumscribed or ill-defined appearances.

Disease classification was conducted according to standard criteria: unifocal (a single lesion in one organ), multifocal (multiple lesions within a single organ system), or multisystem (involvement of two or more organ systems) [1].

Treatment approaches were categorised as conservative (observation with or without biopsy and curettage) for unifocal disease, and systemic chemotherapy using vinblastine and prednisolone according to contemporary LCH-III (before 2017) or LCH-IV (2021 onward) protocols for multifocal and multisystem disease [6].

Patient outcomes assessed included lesion resolution or progression, occurrence of relapse, and overall survival. No skeletal-related complications such as fractures or spinal instability were reported. Complete healing of a bone lesion was defined radiographically by re-ossification or sclerosis of the lytic defect [6].

Descriptive statistics were performed using Microsoft Excel, summarising categorical data as counts and percentages. 

Statistical analysis: All data were analysed descriptively using counts (*n*), percentages (%), and mean ± standard deviation (SD). No inferential statistical tests (such as t-tests, chi-square tests, or analysis of variance (ANOVA)) were performed, as the study did not include hypothesis testing or comparisons between independent groups. Statistical significance thresholds (e.g., *P* < 0.05 or *P* < 0.001) were therefore not applicable.

## Results

Patient demographics and disease extent

Thirty-five paediatric patients with LCH were included, comprising 22 males (62.9%) and 13 females (37.1%). Ages ranged from 19 days to 16 years (mean 5.94, median 4 years). Of the 35 patients, 30 (85.7%) had skeletal involvement, with disease limited to the skeletal system (SS LCH) in 25 patients (71.4%). Within the SS skeletal group, 23 patients (65.7%) had a unifocal bone lesion, and 7 patients (20%) had multifocal bone lesions without extra-skeletal involvement. Five patients (14.3%) had multisystem LCH, with bone lesions accompanied by extra-skeletal involvement including the skin (3 cases), lymph nodes (3 cases), pituitary gland (2 cases), lungs (1 case), liver and spleen (1 case), and bone marrow (1 case). Overall, 12 patients (34.3%) had multiple bone lesions (7 multifocal SS cases plus 5 multisystem cases). The proportion of unifocal to multifocal bone presentations (~66% vs. 34%) is comparable to other paediatric cohorts reported previously (Table 1) [7].

**Table 1:** Demographics and clinical characteristics of paediatric patients with LCH (N = 35). LCH, Langerhans cell histiocytosis; SD, standard deviation; IHC, immunohistochemistry

Variable	n	%
Sex		
Male	22	62.9
Female	13	37.1
Age		
Median age (years)	-	4
Mean age (years ± SD)	-	5.94
Age range	-	19 days–16 years
Disease classification		
Unifocal single-system	23	65.7
Multifocal single-system	7	20.0
Multisystem disease	5	14.3
Skeletal involvement		
Skeletal involvement	30	85.7
Ill-defined lytic lesions	28	93.3 (of skeletal cases)
Well-defined lytic lesions	2	6.7 (of skeletal cases)
Skeletal lesion sites		
Skull	22	62.9
Femur	6	17.1
Scapula	3	8.6
Spine	3	8.6
Pelvis	2	5.7
Sternum	2	5.7
Clavicle	1	2.9
Rib	1	2.9
Humerus	1	2.9
Ulna	1	2.9
Tibia	1	2.9
Extra-skeletal involvement		
Skin	3	8.6
Lymph nodes	3	8.6
Pituitary	2	5.7
Lungs	1	2.9
Liver and spleen	1	2.9
Bone marrow	1	2.9
Immunohistochemistry		
IHC performed	27	77.1
Positive (CD1a/S100/CD68 ± Langerin)	24	68.6
Negative	3	8.6
Clinically diagnosed without biopsy	8	22.9
Genetic testing		
BRAF tested	7	20.0
BRAF V600E	3	8.6
BRAF V600D	1	2.9
Negative	3	8.6
Treatment		
Observation/biopsy/curettage	19	54.3
LCH-III protocol	10	28.6
LCH-IV protocol	6	17.1
Outcomes		
Relapse	8	22.9
Mortality	0	0

Anatomic distribution of skeletal lesions

A total of 43 discrete bone lesions were identified among the 30 patients with skeletal disease. The most frequently affected site was the skull, involved in 22 patients (73.3% of skeletal cases). Skull lesions typically involved calvarial bones (frontal, parietal, occipital) or craniofacial bones; several patients had multiple skull lesions.

**Figure 1:**
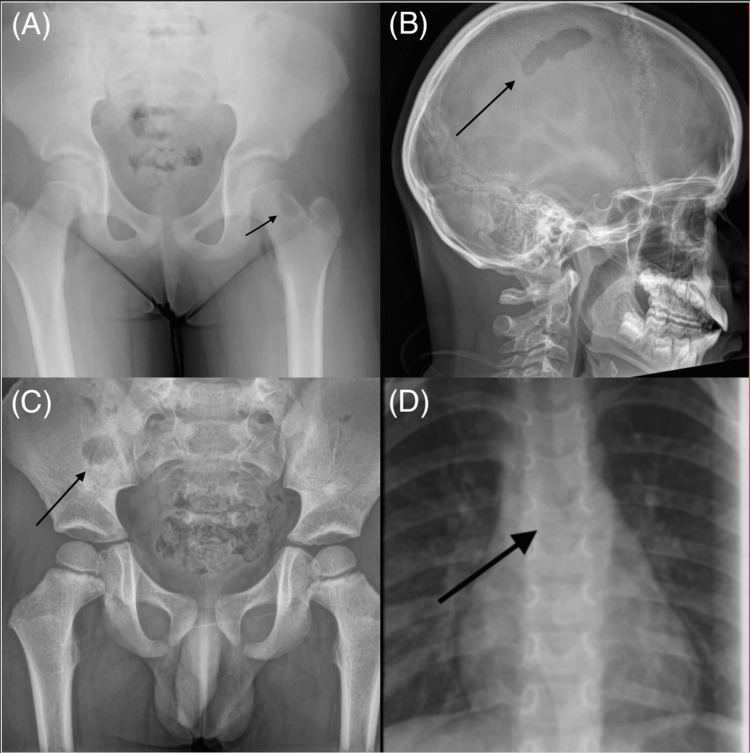
Representative skeletal lesions in paediatric Langerhans cell histiocytosis. (A) Left femoral neck lesion in an 11-year-old child. (B) Left parietal skull lesion in a nine-year-old child. (C) Right ilium lytic lesion in a three-year-old child. (D) Thoracic (T6) wedge collapse in a three-year-old child.

After the skull, the femur was the second most common site, affected in 6 patients (20%), usually involving the proximal region or femoral shaft. This prevalence is slightly higher compared to larger series (~10%), though long-bone involvement, particularly of the femur, is well-recognised [6]. Other affected sites included the scapula (3 patients, 10%), spinal vertebrae (3 patients, 10% - one thoracic, one lumbar, one sacral), pelvis (2 patients, 6.7%), sternum (2 patients, 6.7%), clavicle (1 patient, 3.3%), rib (1 patient, 3.3%), humerus (1 patient, 3.3%), ulna (1 patient, 3.3%), and tibia (1 patient, 3.3%). All bone lesions were purely lytic on plain radiographic imaging. The majority of lesions (28 of 30 skeletal cases, 93.3%) were ill-defined in appearance, reflecting aggressive bone destruction. Two lesions were well-circumscribed, one in the iliac wing of a two-year-old and another in the femoral neck of an 11-year-old. No lesions occurred in the bones of the hands or feet, consistent with prior observations indicating that LCH seldom affects distal extremities (Figure 1) [6].

Management

Treatment strategies were tailored to the extent of the disease and lesion characteristics. SS unifocal bone LCH (*n *= 23) was managed with a focus on local control. 18 of these patients underwent a diagnostic biopsy and curettage of the lesion with no further therapy. In the remaining 5 unifocal cases, the patients were observed without adjuvant therapy. Notably, no unifocal bone patient in our series required systemic chemotherapy, consistent with current practice that solitary bone LCH can often be managed with minimal intervention [5]. SS multifocal bone LCH (*n* = 7) was managed more aggressively: all 7 received systemic therapy with vinblastine and prednisone (the standard first-line regimen [4]), usually for 12 months as per international LCH protocols. Multisystem LCH cases (*n* = 5) were treated with systemic chemotherapy as per risk-adapted protocols. All 5 received vinblastine/prednisone-based therapy.

Outcomes

Overall, outcomes were excellent in this cohort. At the time of the last follow-up, all 35 patients (100%) were alive, with disease either in remission or stable. This survival rate aligns closely with published survival rates approaching 100% for SS LCH in children [5].

Disease relapse occurred in 8 patients (22.9%), though all episodes subsequently resolved. This relapse rate is consistent with previous reports, which document recurrence rates ranging broadly from 10% to over 20% in paediatric LCH [6,7].

Bone Healing and Sequelae

Radiographic healing or remineralisation of bone lesions typically occurred over approximately six months in cases of unifocal lesions and around nine months in cases of multifocal lesions requiring ongoing therapy, consistent with other studies noting similar healing times [6]. There were no documented cases of pathological fractures, spinal instability, or permanent growth disturbances in this cohort. Importantly, no patient was identified as having long-term skeletal or functional impairments related to LCH.

## Discussion

This case series highlights the dominant role of the skeletal system in paediatric LCH. In our cohort, 85.7% had SS (bone-only) disease, reflecting this predilection. Consistent with larger studies, we found that the skull and other axial bones are the most common sites of LCH involvement in children [2,6]. Among patients with skeletal involvement (n = 30), 73.3% had craniofacial lesions. This figure falls within the broad range reported in the literature: for example, an Egyptian series reported ~80% craniofacial involvement among skeletal LCH cases [6], while a review by Choi et al. noted over 50% of LCH bone lesions occur in the skull [2]. The mandible was involved in 10% (3 out of 30 patients with skeletal involvement), consistent with reports that the mandible is the second most frequently affected bone in paediatric LCH, involved in approximately 10% of cases [3]. Long bones of the limbs, especially the femur, were also frequently affected in our series (20% of patients had femoral lesions). Long bone involvement is well-documented in children with LCH and tends to occur more often in the paediatric population than in adults [2]. Our rate of femur lesions is higher than some older series (which noted ~17% of cases with any long bone involvement) [2], but this may be due to random variability in a small sample. Ribs and pelvic bones appeared in about 3%-10% of our cases, again matching known patterns (ribs are reported in ~6-20% and pelvis ~7-13% of patients depending on series) [2]. Spinal involvement was observed in 10% of patients in our series, consistent with the 3-10% range reported in previous paediatric LCH studies [2]. However, the true frequency of vertebral lesions can vary, and some series focusing on bone LCH have found spine lesions in up to 15-20% of cases [6]. Importantly, in children, thoracic vertebrae are the most commonly affected, and lesions often result in vertebra plana (complete collapse of the vertebral body) [8]. The clinical outcome in our spine cases was good, with no neurological problems.

The clinical course of bone LCH in children is generally benign in terms of survival, especially when the disease is SS. In our study, there were no deaths, which concords with large analyses showing near-100% overall survival in patients with LCH restricted to bone and no risk-organ involvement [1]. Even multisystem LCH now has a high survival rate (5-year survival >90% in recent trials) with modern chemotherapy, though it remains less predictable in course [1]. The excellent prognosis for isolated bone LCH should be balanced against the morbidity that lesions can cause - such as pain, pathological fractures, or deformities. About 20-30% of children with SS bone LCH have been reported to develop long-term consequences, mostly orthopaedic in nature (e.g., chronic spinal deformity or limb asymmetry) [1,6]. In our series, none of the patients experienced lasting orthopaedic complications, and all skeletal lesions healed without significant sequelae, reflecting favourable musculoskeletal outcomes. Our findings support the notion that most bone lesions in LCH tend to heal or remodel over time, especially with the regenerative capacity in young children [6]. This spontaneous healing tendency has been noted by other authors as well: Ghanem et al. observed that many solitary bone lesions showed partial or complete regression without aggressive intervention [9].

Therapeutically, our approach and outcomes reflect contemporary standards in LCH management. For unifocal bone lesions, we favoured minimally invasive treatment - biopsy for diagnosis followed by observation or local therapy - over systemic chemotherapy. This approach is supported by the literature, as solitary eosinophilic granulomas of bone often resolve with curettage or even observation alone [5,6]. Systemic treatment (usually vinblastine and corticosteroids for 6-12 months) is typically reserved for patients with multifocal bone disease or multisystem involvement [6]. All patients in our series who met those criteria did receive systemic therapy, and the high response rate is consistent with international trial results [5]. Notably, none of our patients underwent extensive surgical resection of lesions or high-dose radiation, interventions that were used in past decades but are now seldom indicated in LCH due to their potential morbidity and the availability of effective medical therapies [7].

Our study is limited by its retrospective design and relatively small sample size. The cohort size (*n* = 35) is modest compared to multi-institutional studies; hence, some of the percentage comparisons (e.g., frequency of lesions in certain bones) should be interpreted with caution due to wider confidence intervals. Nonetheless, the distribution of skeletal involvement we observed largely mirrors that reported in larger series, lending credibility to our findings. Another limitation is potential referral bias - as a tertiary centre, we might see more complex or multifocal cases, which could skew the proportion of multisystem disease upward. Despite these limitations, the detailed radiologic and clinical follow-up data strengthen our conclusions about outcomes (healing and complications). We also did not formally assess quality of life or functional outcomes beyond noting physical sequelae; future studies could incorporate patient-reported outcomes to better quantify the impact of skeletal LCH on daily life.

In summary, our experience with 35 children reinforces key points about paediatric LCH with bone involvement. First, clinicians should maintain a high index of suspicion for LCH in any child with lytic bone lesions, especially in the skull or long bones, even if the presentation mimics infection or other tumours [5]. Timely biopsy and diagnosis can prevent unnecessary treatments (such as prolonged antibiotics for presumed osteomyelitis) and allow appropriate therapy [5]. Second, the management of bone LCH can often be conservative - many lesions will stabilise or resolve with minimal intervention, and overtreatment should be avoided in unifocal cases to minimise therapy-related toxicity [5]. Third, in cases of multifocal or high-risk disease, systemic therapy according to established protocols yields excellent disease control in the majority of patients, as reflected in our 100% survival and high remission rate. Finally, long-term follow-up is essential, as relapses can occur, and late sequelae (endocrine or orthopaedic) may emerge over time [1,6]. With vigilant monitoring and supportive care, even children who suffer skeletal damage from LCH (e.g., vertebra plana) can lead normal lives, thanks to the remarkable capacity for recovery and remission in this disease.

## Conclusions

Paediatric LCH most commonly involves the skeletal system, often as the sole site of disease. Our 35-patient case series confirms that within the skeleton, the craniofacial bones (skull and mandible) are the predominant sites of LCH lesions, followed by long bones (such as the femur), scapula, spine, and pelvis. Awareness of this distribution can guide clinicians in focused screening for additional lesions (e.g., skull radiographs or skeletal survey) once an index lesion is diagnosed. The prognosis for children with bone-only LCH is overwhelmingly positive - in our series, all patients survived, and the vast majority achieved complete remission with no significant long-term disabilities. Even in multifocal bone disease, outcomes are favourable with appropriate therapy, though careful observation for recurrence is warranted. Modern management strategies emphasise conservative local treatment for solitary bone lesions and systemic chemotherapy for multifocal or multisystem disease, yielding high cure rates while minimising overtreatment. Our findings add to the growing body of evidence that many LCH bone lesions heal spontaneously or with minimal intervention, and that irreversible complications are uncommon when patients receive timely, multidisciplinary care. Ongoing research into targeted therapies (such as BRAF and MEK inhibitors for mutation-positive cases) holds promise for even better outcomes in refractory LCH. In conclusion, clinicians should be reassured that paediatric LCH with skeletal involvement - though it may present dramatically with bone destruction or fractures - has an excellent prognosis. Early diagnosis and stratified therapy are key: by tailoring treatment intensity to disease extent, we can ensure that children with LCH not only survive but also thrive with normal growth and development.
